# A laparoscopic approach for removal of ovarian remnant tissue in 32 dogs

**DOI:** 10.1186/s12917-018-1658-y

**Published:** 2018-11-07

**Authors:** Sebastiaan A. van Nimwegen, Bart Van Goethem, Jeffrey de Gier, Jolle Kirpensteijn

**Affiliations:** 1 Department of Clinical Sciences of Companion Animals, Faculty of Veterinary Medicine, Utrecht University, Yalelaan 108, 3584CM, Utrecht, the Netherlands; 2 Department of Small Animal Medicine, Faculty of Veterinary Medicine, Ghent University, Salisburylaan 133, 9820 Merelbeke, Belgium; 3 Hill’s Pet Nutrition, 400 SW 8th Ave, Topeka, KS 66603 USA

**Keywords:** Ovarian, Remnant, Canine, Dog, Laparoscopy, Celiotomy, ORS, GnRH, Laparotomy, Ovariectomy

## Abstract

**Background:**

Surgical treatment of ovarian remnant syndrome (ORS) in dogs usually necessitates large celiotomies and considerable manipulation of organs because of the relatively deep position of ovarian remnant tissue, large patient size, and often encountered adhesions. In women, laparoscopic treatment of ORS is successful and has significant advantages over laparotomy. Since laparoscopic ovariectomy has significant advantages over open ovariectomy in dogs, including reduced surgical stress and postoperative pain and shorter convalescence period, the rationale for a laparoscopic approach of canine ORS is evident. Feasibility and efficacy of a laparoscopic approach for treatment of ORS in dogs was prospectively evaluated using a standardized protocol for diagnosis, treatment, and follow-up. Treatment success was evaluated by histology of removed tissues, postoperative hormone testing, and long-term clinical follow-up.

**Results:**

Thirty-two client-owned predominantly medium and large breed dogs diagnosed with ORS underwent abdominal ultrasound for ovarian remnant localization prior to laparoscopic surgery for removal of ovarian remnants. Tissue dissection and excision was performed using a vessel sealing forceps. Laparoscopy subjectively enabled detailed visibility and facilitated detection and removal of suspected ovarian tissue in all cases. Histology confirmed ovarian origin of removed tissue in all dogs. Additionally, a GnRH stimulation test was performed in fourteen dogs after a median follow-up of 10.5 months, which verified absence of residual functional ovarian remnant tissue in all dogs. Median surgery duration was 97.5 min and mean total convalescence duration, subjectively scored by owners, was 1.5 ± 0.7 days. No major complications occurred. Adhesions were observed in 79% of the dogs, complicated the surgical approach, and significantly affected surgery duration (85 versus 109 min; *p* = 0.03). Minor hemorrhage occurred in 12% and significantly increased surgery duration (95.5 versus 128 min; *p* = 0.02). Trendelenburg position and lateral tilting of the patient were essential for proper access to ovarian remnants. GnRH stimulation test results and/or absence of clinical signs indicative of ORS after a median follow-up period of 22.5 months confirmed treatment efficacy in all dogs.

**Conclusion:**

Laparoscopic surgery for ORS in dogs is effective with minimal complications and short convalescence and can successfully replace the conventional, more invasive open surgical procedure.

**Electronic supplementary material:**

The online version of this article (10.1186/s12917-018-1658-y) contains supplementary material, which is available to authorized users.

## Background

Ovarian remnant syndrome (ORS) can occur as a complication after ovariectomy (OVE) or ovariohysterectomy (OVH). The syndrome is defined by clinical signs caused by hormone production by ovarian remnant tissue. Incidence rates of 0% (0/135), 0.1% (2/1880), and 0.5% (3/618) have been reported [1–3]. Despite anecdotal causes of ovarian remnant tissue, such as accessory or ectopic ovarian tissue or revascularization of ovarian tissue that dropped in the abdominal cavity during surgery, the only truly proven cause in dogs with ORS is iatrogenic, i.e. not removing the entire ovary during the OVE/OVH procedure [4, 5]. Possible risk factors for canine ORS are associated with accessibility of the ovaries during surgery and include large, deep-chested dogs, obesity, a too small and/or more caudal position of the celiotomy incision (OVH versus OVE) and seem to correspond with a higher incidence of right-sided locations of ovarian remnant tissue [4–9]. Clinical signs from ovarian hormone production usually occur within several months after the initial procedure [7], although they can arise from as early as 1 week to as late as 11 years after the initial surgery [8]. Clinical signs of ORS can include symptoms associated with (pro) estrus, such as behavioral changes, attractiveness for male dogs, swelling of the vulva, serosanguineous vaginal discharge and receptivity to mating. Additionally, cystic endometrial hyperplasia, (stump) endometritis, pseudopregnancy, vaginitis, and ovarian neoplasia [4, 5, 7], and less commonly diabetes mellitus [10] and abdominal pain [11] are described. In contrast, ORS in humans is mainly associated with chronic inflammatory processes causing abdominal pain [12].

Diagnosis of ORS can be reliably made using a combination of history, clinical signs, vaginoscopic findings and vaginal cytology during (pro) estrus, measurement of the plasma progesterone (P_4_) concentration during the expected luteal phase [7, 13]. When results of these tests are not diagnostic but suspicion of ORS remains, a GnRH stimulation test can be used to rule out or confirm the existence of an ovarian remnant [8].

Treatment of ORS consists of surgical excision of all the ovarian remnant tissue. The common approach is via midline celiotomy. After a thorough abdominal exploration, all suspicious tissue is excised, especially near the original OVE/OVH excision sites, as macroscopic distinction between ovarian remnant and fibrous (scar) tissue can be difficult. Because of the dorsal location of the ovarian pedicle and adhesions caused by the previous surgery, careful dissection is necessary to avoid damage to the ureters and other closely associated organs/structures. Performing surgery during estrus-like clinical signs aids the discovery of very small remnants or remnants covered by adhesions because of increased size and vascularization and the presence of developing follicles or *corpora lutea* [5, 8, 14]. Consequently, a large celiotomy incision and manipulation of organs are usually required for thorough exploration of the ovarian pedicle and uterine horn locations. All lesions including cystic abnormalities and foci of abnormal vascularity should be excised and histologically evaluated for ovarian remnant tissue. Recurrence of ORS after surgical intervention is common [15] and referral to a surgical specialist has been advised [16, 17].

Considering that surgery for ORS is accompanied by a larger celiotomy and considerably more manipulation of abdominal tissues than elective ovariectomy or ovariohysterectomy, the rationale for developing a minimally invasive approach was logical. Laparoscopic ovariectomy has become a routine procedure in dogs and cats with multiple studies showing reduced surgical stress response and post-operative pain and faster convalescence compared to an open celiotomy approach [18–25]. In women, laparoscopic treatment of ORS is generally successful [12, 26], has a low recurrence rate (up to 7%) [27–29], and is associated with decreased blood loss, a shorter hospital stay, and fewer postoperative complications compared to celiotomy [30]. Only a few case reports and three small retrospective case series exist of laparoscopic surgery for ORS in dogs and cats [15, 31–34]. Confirmation of full excision of ovarian remnant tissue was often made only by absence of clinical signs.

In the study reported here, a laparoscopic approach for removal of ovarian remnant tissue was prospectively investigated in dogs diagnosed with ORS, using a standardized protocol for diagnosis, treatment, and follow-up, including post-operative confirmation of the absence of any ovarian tissue using a GnRH stimulation test in a subset of 14 dogs. We hypothesized that a laparoscopic approach for removal of ovarian remnant tissue would be feasible in dogs without the need for conversion to a celiotomy, resulting in a high success rate and short convalescence period.

## Materials and methods

### Inclusion criteria

All dogs diagnosed with ORS, between July 2009 and February 2014, at the Department of Clinical Sciences of Companion Animals, Utrecht University and the Department of Small Animal Medicine, Ghent University, were offered a laparoscopic surgical approach for the same costs as a laparoscopic ovariectomy. A research grant by the Dutch Society for Veterinary Research in Companion Animals (Stichting D.O.G.) [35] enabled follow-up GnRH-testing in the first 13 dogs without additional costs for the owners. The study and associated protocol was approved by the local Ethical Committee of the Department of Clinical Sciences of Companion Animals, Utrecht University.

### Diagnostic protocol

For inclusion in the study, ORS had to be proven by a combination of clinical history and clinical examination followed by functional endocrine tests if necessary. Signs of (pro) estrus, if present upon presentation to the clinic, were clinically investigated using vaginoscopy and vaginal cytology. In late follicular phase and suspected luteal phase, based on history and clinical examination, measurement of P_4_ concentration was performed. Normal basal P_4_ in ovariectomized bitches in our laboratory: median, 0.63 nmol/l (range: < 0.15–2.2 nmol/l). A P_4_ concentration of higher than 3.36 nmol/l is diagnostic for ORS [36]. In cases in which these tests were negative, but suspicion of ORS remained, a GnRH stimulation test was performed.

### Ultrasound imaging

Additionally, in all dogs an abdominal ultrasound was performed to search for abnormalities that could be related to ORS and to evaluate the ability to predict the location of ovarian remnant tissue by ultrasound. Ultrasound examination was performed using a C8–5 broadband curved array transducer at 8 MHz with a HD digital ultrasound system (HD11 XE, Philips, the Netherlands). If indicated, adjustment of ultrasound frequency in the individual patient was based on image quality and patient size.

### Anesthetic protocol

Two different anesthetic protocols were used. Healthy ASA 1 and 2 patients received anesthesia according to a routine protocol consisting of dexmedetomidine (Dexdomitor, Orion Corporation, Finland) premedication (10–15 μg/kg IV), propofol (PropoFlo Plus, Abbott Logistics B.V., the Netherlands) induction (1–2 mg/kg IV to effect), and isoflurane (Isoflo, Abbott Laboratories, United Kingdom) in 50% O_2_–50% air mixture as maintenance. Dogs were mechanically ventilated (IPPV) during surgery. Dexmedetomidine was routinely redosed at 5–7.5 μg/kg IV after 60 min of anesthesia. Additional analgesia consisted of carprofen (Rimadyl, Zoetis, België) (4 mg/kg IV) and buprenorphine (Buprecare, Animalcare ltd, United Kingdom) (10–15 μg/kg IV). After surgery, atipamezole (Antisedan, Orion Corporation, Finland) (50–75 μg/kg IM) was administered to reverse dexmedetomidine effects. Routine anesthetic monitoring consisted of esophageal temperature probe, capnography, pulse oximetry, end-tidal O_2_, end-tidal CO_2_, end-tidal isoflurane, ECG, heart rate, respiratory rate, tidal volume, airway pressure, non-invasive blood pressure. Dogs older than 7 years of age, or with increased risk of circulatory compromise received a different anesthetic protocol consisting of methadone (Comfortan, Eurovet Animal Health B.V., the Netherlands) (0.05 mg/kg IV) and midazolam (Dormicum, Roche B.V., the Netherlands) (0.4 mg/kg IV) premedication, followed by propofol induction (2–4 mg/kg IV to effect) and isoflurane in 50% O_2_–50% air mixture maintenance. Additional analgesia consisted of constant rate infusion of fentanyl (Bipharma, the Netherlands) (10–20 μg/kg/hr) during maintenance of anesthesia, which is partially reversed during recovery by administering buprenorphine (10–15 μg/kg) while maintaining adequate postoperative analgesia. Postoperative analgesia consisted of 3 days of oral carprofen (2 mg/kg 2dd). Antibiotics were not part of the treatment protocol and were only sporadically administered as prophylaxis in cases of (partial) hysterectomy of suspected septic inflammation or contaminated contents of the uterus.

### Surgery protocol

No attempt was made to plan the time of surgery to coincide with estrus for improved visualization of remnants. On the contrary, surgery was often postponed until anestrus for dogs that presented with overt estrus and a history of regularly occurring estrous cycles to decrease the chance of postoperative pseudopregnancy. For dogs that did not present with signs of regularly occurring estrous cycles, surgery was usually planned shortly after diagnosis. All surgeries were performed by three experienced laparoscopic surgeons with ECVS board certification (SvN, JK, BVG). The dogs were placed in 10-15^o^ Trendelenburg position with alternate 20-30^o^ lateral tilting. Surgery was performed through a standard 3-portal midline laparoscopic approach; using a modified Hasson approach with 6-mm outer diameter blunt tip threaded screw-in cannulas (Ternamian EndoTIP, Karl Storz Endoscopy, Germany). The first portal was midway between umbilicus and pubic rim. A 10 mm skin incision was made, followed by blunt dissection of subcutaneous tissue, revealing the linea alba. Two stay sutures were placed in the external abdominal fascia, bilaterally at 2 mm from the linea alba. The stay sutures were pulled up and a 4–5 mm stab incision was made in the linea alba. The cannula was placed under visual guidance of a 5 mm 0^o^ or 30^o^ telescope (Hopkins II, Karl Storz Endoscopy, Tuttlingen, Germany) inserted in the cannula [37]. After visual confirmation of proper abdominal entry, the abdomen was insufflated with CO_2_ gas to an intra-abdominal pressure of 8 mmHg (Thermoflator, Karl Storz Endoscopy, Tuttlingen, Germany). Two additional 6-mm portals were placed 1–2 cm caudal and 1–2 cm cranial to the umbilicus, respectively. The telescope was then inserted through the middle portal and a routine inspection of the abdomen was performed. Surgery was commenced according to a preplanned routine: inspection of the left ovarian pedicle location and uterine remnant (if present) with the dog tilted to the right side; inspection of the right ovarian pedicle location and uterine remnant (if present) with the dog tilted to the left side; inspection of the cervical stump (if present). As a rule, adequate access to the ovarian pedicle area included at least proper visualization of both kidneys. Furthermore, any abnormalities and omental adhesions were inspected. In cases where remnants were not visually identified, existing omental adhesions were carefully inspected and followed towards any attachment site. Likewise, the round ligament of the uterus was identified at the inguinal ring and followed cranially. Any suspect looking tissues, adhesions, cystic structures or ligature scars were excised from the pedicle region and any cystic or inflammatory changes of the uterine remnant or locations of omental adhesions to the uterine remnant were also excised. A fourth portal for extra tissue manipulation was used if necessary. Manipulation of tissues was routinely performed using 5 mm diameter endoscopic instruments, including a Kelly dissection forceps and a self-retaining blunt grasping forceps. Additional instruments for tissue manipulation were optional, including fan retractors, Babcock forceps, endoscopic suction/irrigation device, and endoscopic scissors. All tissue dissection and excision was performed using a vessel sealing device (VSD; LigaSure, Covidien, Boulder, CO). Excised tissues were removed from the abdominal cavity either through a 10-mm cannula that was exchanged for the 6-mm cannula in the caudal portal, or through an enlarged caudal portal. Larger tissue specimens or fragments were removed using a 10-mm specimen retrieval bag. Uterine remnants with hormone-induced changes within the normal range were not excised. In cases of abnormal uterine changes, such as overt cystic endometrial hyperplasia or pyometra, the uterine remnants were excised intra-abdominally using the VSD, or laparoscopy-assisted hysterectomy was performed with removal of the uterus through an enlarged caudal portal and either ligated and transected with scissors or transected using VSD outside of the abdomen. Isolated uterine abnormalities, such as (para-) uterine cysts or adhesions were treated by local excision of the abnormal tissue. Closure of portals larger than 6 mm was done in 3 layers (external abdominal fascia, subcutis, and skin) using absorbable monofilament suture material. In portals < 6 mm where the external abdominal fascia was not easily accessible, only subcutis and skin were closed. Dogs were released from the hospital directly after recovery from anesthesia and owners were instructed the keep their dog on a leash with restricted exercise for 3–5 days.

### Follow-up protocol

Owners were routinely contacted by telephone after 1 week to inform them of the histology results and a standardized questionnaire was used to obtain information on the immediate postoperative period, scoring the duration of convalescence (how long it took until the dog was acting as before the operation, seemingly not affected by the surgery or anesthesia, as subjectively scored by the owner), occurrence of pseudopregnancy, occurrence of wound complications, changes in appetite, behavior, and activity levels of the dog, and owner satisfaction with the treatment and outcome. For the first 13 dogs, a routine control visit was planned, preferably more than 3 months post-operatively, during which a GnRH stimulation test was performed in order to evaluate if all ovarian remnant tissue was removed during surgery. After the initial 13 cases, post-operative GnRH testing was only performed if clinical signs of possible recurrent ORS were suspected. Long-term follow-up consisted of several telephonic contact moments with the owners throughout the duration of the study, scoring any behavioral or clinical signs suggestive of ORS, and monitoring changes in appetite and activity level. Short- and long-term follow-up questionnaire forms were added as an additional file (see Additional file 1).

### GnRH stimulation test

Blood samples were collected at − 40, 0, 10, 60, and 120 min by venipuncture. At *t* = 0, immediately after collecting the second blood sample, a GnRH analogue, 10 μg/kg body weight Gonadorelin (Fertagyl; Intervet/Schering-Plough Animal Health, Boxmeer, the Netherlands) was administered intravenously. Plasma hormone concentrations were measured as previously described [8], at the following time points: estradiol-17β (at − 40, 0, 60, and 120 min), LH and FSH (both at − 40, 0, 10, and 60 min). Before GnRH stimulation at *t* = 0, baseline hormone levels were measured twice with 40 min time interval and values were averaged for increased accuracy [8]. Previously established cut-off values were used to differentiate between dogs with and without residual ovarian tissue (Table 1) [8, 38]. LH measurements after GnRH stimulation, at 10 and 60 min, were routinely performed to confirm that GnRH stimulation was effective as indicated by a short-lived increase in plasma LH concentration at *t* = 10 min [39]. FSH measurements at t = 10 and 60 min were performed as an extra indication of the absence of functional ovarian tissue as indicated by no further increase in plasma FSH concentration [38].

**Table 1:** Reference values for interpretation of GnRH stimulation test results after removal of ovarian remnants [8, 38]

plasma parameter	time point	cut-off value (ovariectomized)
LH	Basal (before GnRH)	> 3.4 μg/l
FSH	Basal (before GnRH)	> 11.0 μg/l
FSH: Estradiol-17β ratio	Basal (before GnRH)	> 0.66
Estradiol-17β	120 min after GnRH	< 21.3 pmol/L
Estradiol-17β	Basal (before GnRH)	< 7 pmol/l → no ORS 7–21.3 pmol/l → not diagnostic

Time point is relative to the administration of GnRH during a GnRH stimulation test. An animal was considered completely ovariectomized if the measured parameters were > or < the presented cut-off values as specified

### Histologic evaluation

Histologic evaluation was performed through standard H&E stained sections of paraffin-embedded tissue labeled according to their location in the abdomen. In case of negative findings (i.e. fatty tissue without clear evidence of either suture material, inflammatory changes, or scar tissue, or with evidence of mesosalpinx-like structures or cystic structures without ovarian components), additional deeper sections were performed to rule out the possibility of skipping small ovarian remnants.

Initial diagnosis, ultrasound-predicted location, and treatment success were ultimately confirmed by histologic examination of all excised tissues.

### Statistical analysis

Statistical analysis of the data was performed using IBM SPSS 23 data analysis software. Apart from general descriptive statistics, differences between sub-groups were evaluated using Student’s t-test or Mann-Whitney U test if data were not normally distributed, as tested using Shapiro-Wilk test. Statistical relations or trends were evaluated using ANOVA and linear regression tests. Statistical significance was assumed when *p* < 0.05. Results are reported as means ± sd or median (Inter Quartile Range (IQR) and/or range).

## Results

### Patient population

Thirty-two dogs with ORS were included in the study. Mean age was 4.5 ± 2.7 years; mean body weight was 26.7 ± 12.4 kg (range 4.5–71.6 kg). Breeds included 12 (38%) crossbreed dogs, 8 (25%) Labrador Retrievers, 2 German Shepherds, 2 English Cocker Spaniels, a Labradoodle, Airedale Terrier, Boerboel, Dachshund, Flatcoated Retriever, Galgo Espangol, Rottweiler, and a Siberian Husky. Seventy-five percent of the patients had initially been spayed in the Netherlands and Belgium (countries in which the study was performed), 25% of the dogs were previously operated abroad, including Bulgaria, Greece, Turkey, Spain, and the USA. According to the clinical history, the initial surgery consisted of ovariectomy in 59%, and ovariohysterectomy in 31%, and was unknown for 10% of the dogs.

The exact date of initial surgery was known for 18 animals and the median interval between initial surgery and clinical signs of ORS was 8.5 months, (range 0.5–96 months; *n* = 18). Clinical signs that were most frequently reported by the owners were attractiveness to male dogs (96%), vulvar discharge (83%), and vulvar swelling (57%). Less frequently, swelling of mammary glands, occurrence of pseudopregnancy, decreased appetite, frequent urination, and weight loss were reported. Two dogs suffered from increased stress/anxiety that the owners attributed to the ORS. Abdominal pain was present in one dog, pain during defecating in one dog, occasional abdominal discomfort and vomiting in one dog, and a mammary tumor in another. In 6 dogs (19%), treatment of the ORS by exploratory celiotomy had already been attempted by the referring veterinary practitioner. The presence of residual ovarian tissue was confirmed by observation of overt clinical estrus, as confirmed by vaginoscopy and vaginal cytology or distinct endometritis in 56% of dogs. Clinical presentation and measurement of a plasma progesterone level of > 3.36 nmol/l confirmed ORS in 38% of the patients. The remaining 6% was solely diagnosed by means of a GnRH stimulation test.

### Preoperative ultrasound findings

Ultrasound imaging was performed in 31/32 (97%) of the dogs. One dog did not undergo ultrasound imaging because the referring veterinarian knew that a right ovarian remnant was left behind during the ovariectomy procedure but had been unable to locate it and remove it at that time. Ultrasound evaluation indicated at least one suspect tissue in 30/31 dogs. Based on ultrasound evaluation a possible ovarian remnant was located only at the left ovarian pedicle area in 4/31 (13%), only at the right ovarian pedicle area in 16/31 (52%), and at both sides in 10/31 (32%) of dogs. No suspect tissue was found in one dog (3%). Uterine tissue was identified in 26/31 (84%) of the patients, which was abnormal in 14/26 (54%) of the cases (i.e. cystic, enlarged, fluid filled, or signs of endometritis). One coincidental finding was the absence of a left kidney in the presence of a left ovarian remnant in a Labradoodle that was spayed at the age of 8 weeks.

### Surgical findings

**Fig. 1:**
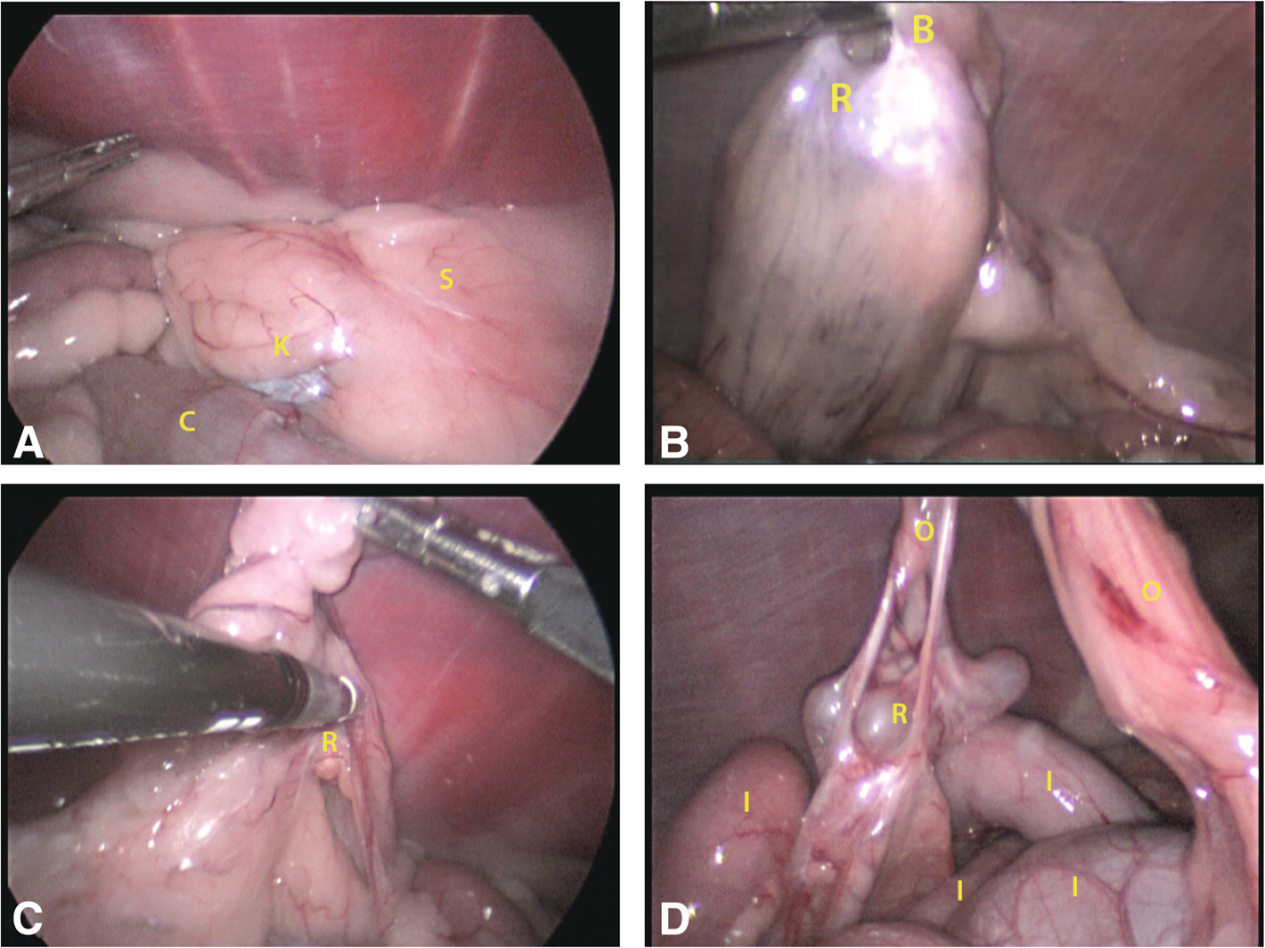
Laparoscopic images of a normal ovarian pedicle scar and several examples of ovarian remnants. 1-**a**: Normal ligature scar in left ovarian pedicle area. 1-**b**: Large left-sided ovarian remnant with forceps in ovarian bursal opening. 1-**c**: Small right-sided ovarian remnant covered by fat and omental adhesions. 1-**d**: Right-sided ovarian remnant with omental adhesions and multiple cystic structures. S = scar; R = ovarian remnant; O = omentum adhesion; K = kidney; C = colon; I = intestine; B = bursal opening

All dogs successfully underwent laparoscopic surgery for ORS without the need for conversion to an open approach. Median duration of surgery, from skin incision to completion of wound closure, was 97.5 min (IQR 24.5; range 60–204 min). Adequate exposure of ovarian remnants sometimes necessitated tilting the dog > 30^o^ laterally or up to 20^o^ in Trendelenburg position. All suspect tissues were excised, including ligature scar tissue, especially if any adhesions, apparent vasculature, or cyst-like changes, even if only very small, were present. Ligature scars with only minimal fibrotic changes without apparent vasculature or other changes were not excised (Fig. 1).

Suspect or abnormal looking tissue was removed from the left pedicle area in 69%, from the right pedicle area in 91%, from the tip of the left uterine horn in 50%, and from the tip of the right uterine horn in 47% of dogs. Removal of the uterine body was performed in 9% of the dogs. The mean number of excised tissue specimens per dog was 2.5 ± 0.9. The size of excised tissues varied from < 1 cm to cystic structures of 5–6 cm in diameter.

**Fig. 2:**
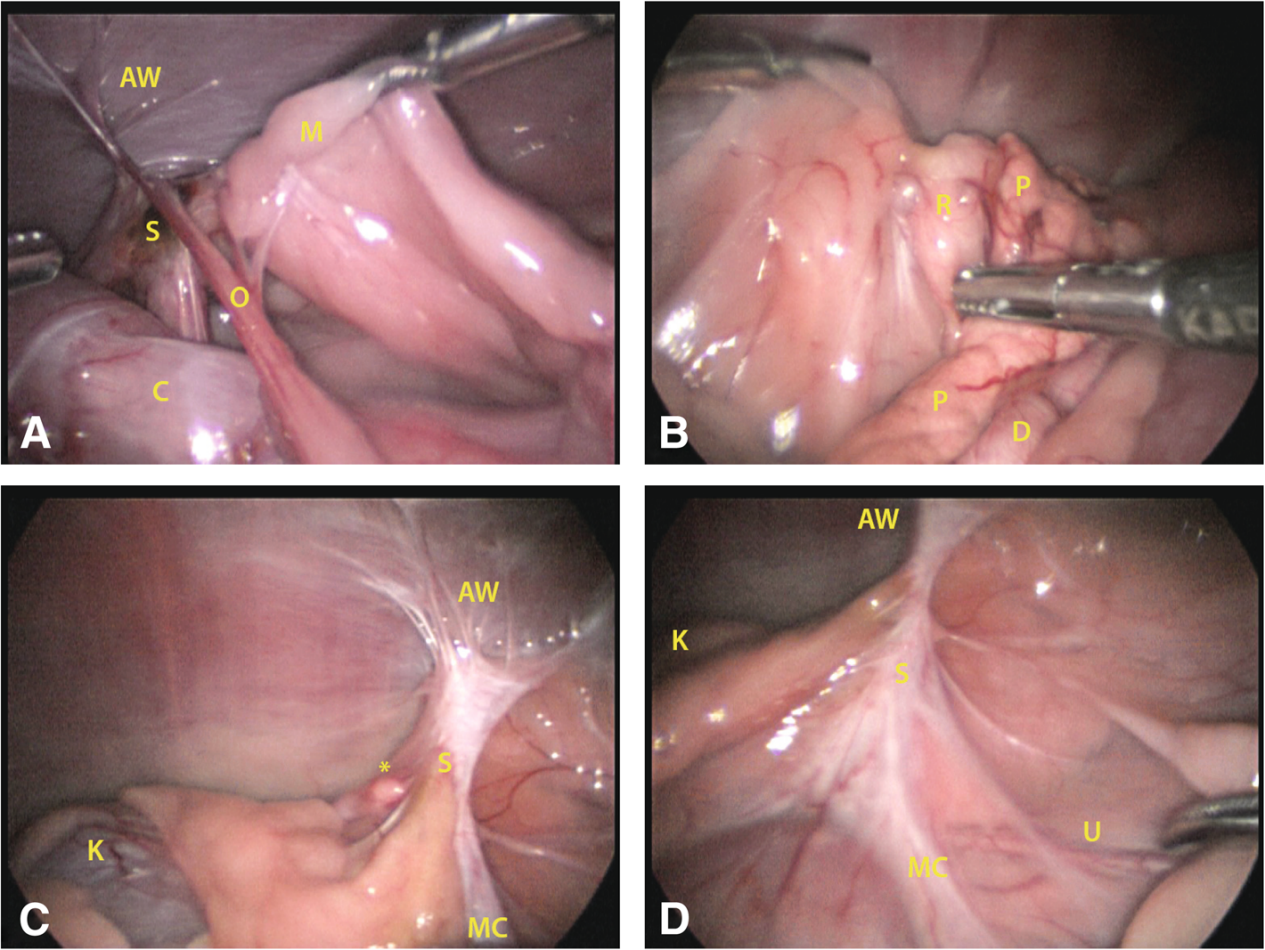
Examples of adhesions in the ovarian pedicle area and visualization of the ureter. 2-**a**: Left-sided scar tissue with multiple adhesions, including abdominal wall. 2-**b**: Right-sided small cystic ovarian remnant adhered to mesoduodenum and pancreas. 2-**c**: . Left-sided ligature scar tissue with multiple adhesions, including abdominal wall and mesocolon. The suspicious looking nodular tissue* turned out to be reactive scar tissue on histologic evaluation. 2-**d**: The same ligature scar tissue as in Fig. 1C, visualizing the left ureter close to ovarian pedicle area. S = scar; R = ovarian remnant; O = omentum adhesion; K = kidney; C = colon; AW = abdominal wall adhesions; MC = mesocolon adhesions; M = mesometrium; P = pancreas; D = duodenum; U = ureter

Adhesions were found in 79% of the dogs (Fig. 2). Adhesions were located in the area of the left pedicle in 41%, in the area of the right pedicle in 67%, to the uterus in 48%, and to the mesoduodenum in 25% of the cases. Surgery duration was significantly faster in dogs without adhesions (median 85 (IQR 41.3) minutes, 18% of dogs) versus dogs with any form of adhesions (median 109 (IQR 36) minutes; 82% of dogs; *p* = 0.03). A fourth, paramedian, portal was placed in 2 dogs for improved access to the surgical area.

### Complications

No major complications occurred. Minor complications consisting of intraoperative hemorrhage occurred in 4 dogs (12%). In one dog, hemorrhage occurred from a paramedian abdominal wall portal which was eventually controlled using a full-thickness abdominal wall suture. Minor bleedings occurred during tissue manipulation in 3 dogs: a splenic puncture by an instrument, several small bleedings occurring during dissection in the right pedicle area, and minor bleeding during dissection of extensive adhesions in the left pedicle area, respectively. All hemorrhages were successfully controlled laparoscopically, using either suction/irrigation or the application of a surgical gauze through a 10 mm portal to improve visibility and establishing hemostasis by plugging the splenic puncture site with a hemostatic gelatin sponge (Spongostan, Ethicon Inc., Somerville, NJ), or the application of the VSD. Total blood loss was considered minimal but the extra effort of evaluation and intervention caused a significant increase in surgery duration in cases of bleeding during ovarian remnant removal compared to non-bleeding cases (median 128 min (IQR 71.5) versus 95.5 (IQR 22) minutes respectively, *p* = 0.02).

Additional surgical procedures were performed in three dogs. One dog had a laparoscopic liver biopsy because of an increased level of circulating bile acids. Another dog had a partial mastectomy because of a concurrent mammary tumor. This turned out to be mammary carcinoma with lymph node metastasis and this dog died 3 months later because of generalized metastasis. A third dog (39 kg cross-breed) turned out to have a sub-clinical gastric volvulus of 270^o^ with moderate dilation, which became apparent during routine laparoscopic inspection of the abdomen. This condition was corrected by deflating the stomach through gastric intubation and laparoscopic correction of the stomach position, followed by a laparoscopic-assisted gastropexy.

### Histology results

Histologic evaluation identified removal of ovarian remnant tissue in all dogs. Ovarian remnant tissue was present only at the left ovarian pedicle area in 3 dogs (9%), only at the right ovarian pedicle area in 23 dogs (72%) and on both sides in 5 dogs (16%). Right-sided remnants were significantly overrepresented (*p* = 0.01). One dog (3%) had an ovarian remnant attached to the uterine horn without involvement of a pedicle area. In this dog, the tips of the uterine horns were adhered to each other and to omentum with a small ovarian remnant within the adhesions.

Several ovarian remnants contained small to large (up to 3 cm) cysts and/or areas representing corpora lutea. In two dogs (6%), the ovarian remnant contained a granulosa cell tumor. Uterine abnormalities consisted of cystic endometrial hyperplasia, endometritis, or localized large cysts (up to 6 cm).

When combining the results of preoperative ultrasound evaluation with the results of histology of excised tissues, the overall accuracy of abdominal ultrasound to predict the location of an ovarian remnant had a sensitivity of 94% and a specificity of 74%. Sensitivity and specificity were also determined for right-sided and left-sided remnant location apart (Table 2).

**Table 2:** Diagnostic accuracy of abdominal ultrasound to locate ovarian remnants in dogs

Ultrasound diagnosis	Sensitivity (95% CI)	Specificity (95% CI)
Left pedicle area	88% (48–99%)	70% (47–87%)
Right pedicle area	96% (81–99%)	100% (n.a.)
Overall	94% (81–99%)	74% (54–99%)

*95% CI* 95% confidence interval

### Postoperative parameters

#### Short-term follow-up

**Fig. 3:**
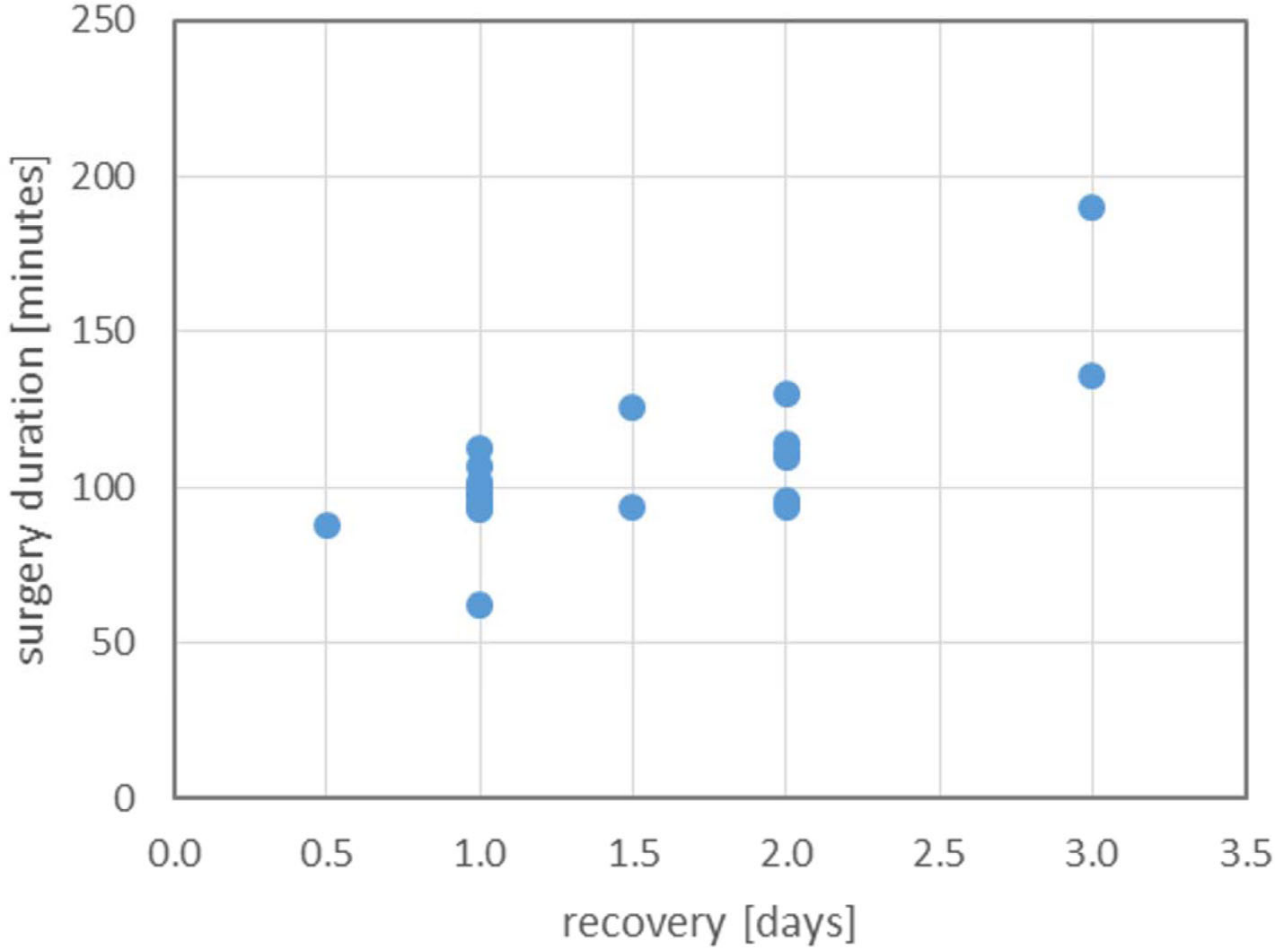
Relation between recovery and surgery duration. Longer surgery duration was significantly related to an increased recovery duration, as subjectively scored by owners

Median time interval from surgery to first follow-up contact with the owners was 7 days (range 3–17 days). Mean duration of recovery, as concluded from the telephone questionnaire with the owners, was 1.5 ± 0.7 days. Recovery duration significantly increased with increased surgery duration (linear regression, Fig. 3, *p* < 0.01). Pseudopregnancy was noted postoperatively by the owner in 4 dogs (12.5%) and resolved without medical intervention. Mild wound healing complications (minor swelling and serous discharge) were seen in 4% of the cases, without requiring medical attention. In the short term follow-up (during the first 2 months postoperative) 3 dogs (10%), one of which was also pseudopregnant, still showed increased attractiveness for male dogs. Another 3 dogs (10%) had an episode of minor vaginal discharge ranging from a white to bloody aspect. Owners reported to be very satisfied to have chosen the laparoscopic approach in 91% of the cases (range satisfied – very satisfied).

#### GnRH stimulation test

GnRH stimulation testing was performed in 13 dogs without clinical signs of ORS. One additional dog was tested because of the occurrence of mild vaginal bleeding 11 months after surgery for ORS. No other clinical signs were apparent in this dog and the only abnormality found during clinical evaluation was a small vaginal septum remnant on vaginoscopy. Median time interval between ORS surgery and GnRH stimulation test was 10.5 months (range 1–25 months). Results of these tests confirmed that ovarian remnant tissue was not present after surgery in all tested dogs. Moreover, none of the measured blood plasma hormone levels could indicate the presence of ovarian tissue, while at least 2 measurements (median 4, range 2–5) per dog indicated the absence of functional ovarian tissue (Table 3). A peak in plasma LH concentration at *t* = 10 min confirmed effective GnRH stimulation in all cases.

**Table 3:** Results of GnRH stimulation tests

Dog case nr.	interval OR - GnRH test [months]	basal E_2_ [pmol/L]	E_2_ *t* = 60	E_2_ *t* = 120	basal LH [μg/L]	LH t = 10	LH t = 60	basal FSH [μg/L]	FSH t = 10	FSH t = 60	basal FSH/E_2_
1	20	18.4	18.0	**16.5**	**25.3**	38.1	22.2	**24.2**	19.0	24.8	**1.31**
2	17.5	19.4	8.8	**8.6**	**19.8**	30.8	25.4	**31.0**	21.6	32.1	**1.60**
3	16.5	9.8	9.2	**11.2**	**11.3**	26.7	13.9	**15.1**	14.3	15.6	**1.54**
4	16	16.5	14.0	**17.6**	**10.1**	26.5	15.1	**11.3**	8.2	13.2	**0.68**
5	18	17.1	12.5	**14.3**	**16.6**	29.0	20.5	**20.6**	21.3	24.0	**1.20**
6	25	**3.2**		**4.5**							
7	12	**2.5**	**1.2**	**0.4**	**15.7**	22.2	12.2	**18.1**	18.3	17.5	**7.22**
8	6.3	**3.9**	**5.9**	**17.3**	**5.9**	10.7	6.6	**13.7**	12.7	15.1	**3.52**
9	9	**4.3**	**2.5**	**2.5**	**19.3**	36.0	15.4				
10	1.5	**3.7**	**1.5**	**0.6**	**14.7**	33.2	15.6	**14.4**	16.3	17.7	**3.88**
11	8.5	**1.8**	**6.4**	**2.0**	**20.8**	43.9	26.6	**20.0**	21.4	20.5	**11.08**
12	3	**3.7**	**3.7**	**5.9**	**10.4**	31.6	8.2	**13.2**	10.8	13.3	**3.56**
13	1	**2.4**	**1.9**	**0.3**	**11.6**	27.8	12.4	7.1^a^	7.9	8.1	**2.95**
14	7	**4.6**	**3.2**	**2.2**	**4.9**	13.1	7.2				

Basal plasma values of estradiol (E_2_), LH and FSH are the average of two separate basal measurements at *t* = −40 and t = 0 min. All measured plasma values that indicate absence of functional ovarian tissue are displayed as bold text, based on previously published cut-off values (see Table 1). None of the values are consistent with the existence of functional ovarian tissue (see Table 1 for cut-off values). ^a^The basal FSH of dog nr. 13 is below cut-off value which in this case was caused by a short time interval between surgery and GnRH stimulation test (see discussion section)

#### Long-term follow-up

Median duration between surgery for ORS and last telephone follow-up was 22.5 months (range 6–53 months). After a follow-up period of 51 months, one dog remained to show increased attractiveness to male dogs according to the owners. This, despite not showing any other signs of ORS and having a negative GnRH stimulation test at 16 months postoperative. None of the other dogs, including the ones that were attractive to male dogs or had vaginal discharge in the short-term follow-up, had any signs of possible ORS in the long-term follow-up. Only one dog had no long-term follow-up because it died due to metastasis of mammary carcinoma 3 months after surgery. Two dogs with reported stress/anxiety, one with abdominal pain, and one with pain during defecation preoperatively had all markedly improved. In one dog, signs of occasional abdominal discomfort and vomiting preoperatively did not improve postoperatively, although the dog became more active and had a better appetite. Signs of ORS disappeared completely after surgery. Overall activity level was subjectively increased in 16% of the dogs, appetite had increased in 16% and was reduced in 1 dog compared to the preoperative situation.

## Discussion

Ovarian remnant tissue was successfully removed by laparoscopic surgery in 32 dogs diagnosed with ORS. All had histologically confirmed ovarian tissue excised. All follow-up GnRH stimulation test results were consistent with the absence of ovarian tissue and all, but one dog, were completely free of clinical signs associated with ORS in the long-term follow-up.

The incidence of referral and type of dogs with ORS in the present study is comparable to previous studies [7, 8]. In a study by Okkens et al. [7], 76% of dogs weighed more than 20 kg which is similar to 75% weighing over 20 kg in the present study, supporting the theory that ORS is more often present in large dogs. Likewise, the significant overrepresentation of right-sided remnants in the present study population is also consistent with other reports of ORS in dogs and may be related to the deeper and more cranial position of the right ovary making it more difficult to access during a standard midline laparotomy approach, which theoretically may increase the risk for ORS [4, 5, 7, 9]. Furthermore, ovarian remnant tissue was found in the pedicle area in 97% of the dogs, which makes other causes for ORS besides surgical error unlikely. There might theoretically be an increased risk for ORS after OVE compared to OVH because OVE necessitates 2 incisions near the ovary (ovarian pedicle and proper ligament) versus only one incision for OVH (ovarian pedicle) [40]. The majority of dogs (59%) in the present study was ovariectomized, which is the most common surgical neutering procedure for female dogs in the Benelux area. Only one dog (3%) had an ovarian remnant attached to a uterine horn, making transection of the proper ligament as a risk factor for ORS less likely. An improper general surgical technique to remove ovaria due to either insufficient visibility or a minimalistic approach seems to be a more likely cause than the discussion surrounding removing the uterus or not as a reason for ORS [40].

A relatively high percentage of dogs (25%) was imported to the Netherlands from other countries and was originally spayed abroad. Although this has not been mentioned in other studies, it may indicate a much higher occurrence of ORS in imported dogs of certain regions. The fact that 20 % of the dogs already underwent surgery by a general practitioner in an unsuccessful attempt to treat the ORS before referral, stresses that surgery for ORS can be a challenge, especially in less experienced hands [16, 17]. The absence of a left kidney in the presence of an intact left-sided ovarian remnant in one dog in the present study that underwent initial spay surgery at the age of 8 weeks may indicate a grave surgical error: the accidental removal of a kidney during initial gonadectomy in this dog. The presence of a single solitary kidney or kidney agenesis cannot be fully ruled out in this dog, however. The absence of a kidney in a dog with ORS has been reported previously [4].

The laparoscopic approach was very effective for abdominal exploration and detection of ovarian remnants or suspect tissues. Being able to get a detailed close up image of selected tissues was subjectively considered advantageous over open surgery for ORS, in which detection and surgical access to an ovarian remnant can be very challenging. Access to the pedicle area was usually considered sufficient. Proper visualization, including visual access to the caudoventral aspect of the kidney, was in most cases achieved using the standard 20-30^o^ lateral tilting and 10-15^o^ Trendelenburg positioning of the patient. This also facilitated identification of the caudal part of the ipsilateral ureter in most dogs, which was helpful for dissection of remnants located near the level of the peritoneum in this area. Difficult access to the pedicle area was mainly caused by the existence of adhesions that would sometimes prevent the intestines, pancreas, or spleen to be dissected away from the area of interest. These cases were managed by increasing the Trendelenburg angle (up to 20^o^) and careful manipulation of adhesions. In two cases, an extra instrument portal was needed for additional retraction of organs to increase visibility of the area of interest. Most of the ovarian remnants could be revealed by locating, grabbing and lifting up the adhered ligaments and omentum. Many of the ovarian remnants were also retracted to some extent and could be recognized by their increased vasculature or bulky or cystic appearance. Gently lifting the ovarian remnant tissue allowed dissection from all surrounding attachments to ligaments, organs, omentum, and retroperitoneal fat. The greatest difficulty was experienced with small ovarian remnants extensively covered by adhesions. In a few cases, the remnant was located in a very dorsal, almost retroperitoneal position. In such ‘retroperitoneal’ cases, the remnant would be covered by fat and could not be manipulated by lifting adhered tissues but would be buried beneath them. Careful probe palpation and dissection was then applied to reveal and remove the ovarian remnant tissue without leaving parts behind or damaging the ureter, which often ran close to the plane of dissection. Adhesions to the mesoduodenum, which were observed in 25% of the cases, could also be challenging because of close proximity of pancreas and pancreaticoduodenal blood vessels.

The amount of adhesions observed in these ORS patients was subjectively considered to be relatively high. Most severe adhesions were usually associated with the ovarian remnant tissue, although the contralateral scar and uterine remnants were equally affected in several cases. ORS may cause or promote chronic inflammatory lesions of the female canine reproductive tract, such as stump pyometra, cystic endometrial hyperplasia, and cystic changes in the ovarian remnant itself [4, 5, 7], which all may contribute to formation of adhesions. In humans, ORS is usually associated with extensive adhesions. However, abdominal inflammatory lesions, mainly endometriosis, are pre-existing to the original oophorectomy in most human patients, and recurrence of these lesions are associated with the ovarian remnant [41], which is not the case in dogs.

The significant relation between surgery duration and postoperative convalescence duration has not been described before. In humans, postoperative convalescence is mainly affected by postoperative pain [42], which seems affected most by pneumoperitoneum pressure level through stretching of peritoneum and diaphragm and possibly chemical irritation of CO_2_ gas [43]. One could argue that prolonged pneumoperitoneum might also increase postoperative pain and convalescence period in dogs. This was however not an objective of the present study and no attempt was made to quantify post-operative pain. Nonetheless, recovery duration was short and comparable to that of laparoscopic ovariectomy [44, 45].

Using a VSD for dissection of adhesions and excision of tissues was convenient and very effective. The VSD was often also used as a mechanical dissecting forceps for dissecting and sealing of tissues with only minimal hemorrhage. Because ovarian remnants may be just a few millimeters in size, we suggest to remove all abnormal tissue that has some noticeable vasculature, small cyst-like changes, clear discoloration, or adhesions.

The diagnostic value of preoperative ultrasound was higher in the present study than previously reported [4, 8]. However, ultrasound alone cannot be used to prove or rule out the existence of functional ovarian tissue. The variation in results in Table 2 between the left and right ovarian pedicle area is probably caused by the relatively low numbers of ovarian remnants on the left side. The relatively low value for overall specificity (74%) of ultrasound diagnosis of the position of an ovarian remnant is largely explained by the high numbers of false positive diagnoses for the left ovarian pedicle area. This may be due to a much lower incidence of ovarian remnant tissue on this side making the chance of false positive diagnosis higher for the left side than for the right side. On the other hand, false positive diagnosis is also affected by detection of scar tissue because ultrasound may not be able to reliably differentiate between scar tissue and an actual ovarian remnant in all cases. Since scar tissue can often not be reliably differentiated from small ovarian remnants by the naked eye and is therefore removed during surgery, the detection of a pedicle scar with ultrasound is considered helpful for ORS surgery. Knowing the location and approximate size of abnormalities seen with ultrasound may help deciding when to stop or go on with searching for abnormalities, especially in patients with extensive adhesions or obesity where only a small remnant was found during surgery. A thorough presurgical evaluation using ultrasonography will help in determining the location and number of abnormalities and thus the surgical strategy. We suggest to always have an ultrasound performed before surgery to lower the risk of an unsuccessful ‘goose’ hunt.

Histology proved that ovarian remnants were removed from all dogs in the present study. Sizes of remnants varied from several millimeters up to complete ovaries and cystic changes of several cm’s. Occasionally, large uterine cysts (up to 6 cm) were encountered, possibly caused by retention of fluid in the uterine lumen cranial to the site of ligation of the uterine horn. Two ovarian remnants turned out to contain a granulosa cell tumor. Formation of granulosa cell tumors in ovarian remnants is relatively common [4, 36]. The true incidence and/or causes of tumor formation in ovarian remnants are unknown. It has been suggested that increased gonadotropin levels may increase the risk of ovarian tumor formation in humans. The same may be true in dogs [36].

All results of follow-up GnRH stimulation tests were consistent with absence of ovarian tissue. These findings were confirmed clinically by the absence of clinical signs of ORS in all, but one, dogs after a median follow-up period of 22.5 months. This one dog continued to show increased attractiveness to male dogs according to the owners. However, the dog was tested negative by GnRH stimulation test 16 months postoperative, was herself not interested in male dogs, and was free of any other sign of ORS after a follow-up period of 51 months. Therefore, attractiveness to male dogs in this animal is most probably caused by another reason. Follow-up clinical investigation other than a GnRH stimulation test was however not performed in this dog.

There was an apparent trend of higher mean basal estradiol values in the GnRH stimulation test results in the early cases (Table 3). These cases also had a longer interval between surgery and GnRH stimulation test. A certain low basal plasma estradiol concentration is normally seen in dogs after gonadectomy, which might be a consequence of endogenous estradiol from conversion of androstenediol from the adrenal cortex [46]. Although not reported before, this process may change in time after surgery, as is the case for LH and FSH plasma levels [38, 47]. Nonetheless, estradiol did not increase after GnRH administration and was below the cut-off value at all times as expected after OVE/OVH (Table 3). The basal FSH of dog nr. 13 could in theory fit with an intact dog (< 11.0 μg/l) [38]. However, more importantly, basal plasma estradiol was low and both FSH and estradiol levels did not increase after GnRH administration, which is consistent with absence of ovarian tissue. It has been described that gonadotrophic hormones are decreased between 4 to 10 weeks after OVE/OVH, after which they steadily increase until week 42 [47]. This is one of the reasons that we preferably performed GnRH stimulation tests after a longer time interval and may explain the low basal plasma FSH in this dog, since it was tested only 1 month after surgery.

### Study limitations

This study prospectively evaluated the feasibility and efficacy of laparoscopic ovarian remnant removal in dogs. The laparoscopic approach provided a clear and detailed visibility of abdominal tissues combined with a short recovery period. However, several intraoperative observations were subjectively scored and because of the lack of a control group, statistical comparison with an open celiotomy approach was not possible.

Several follow-up data were subjectively scored by owners by means of telephone questionnaires, which may have been subject to bias and owner opinion.

## Conclusion

Findings in the study reported here support the theory that ORS is caused by surgical error, with an overrepresentation of right-sided ovarian remnants in predominantly medium and large sized dogs.

A laparoscopic approach for the removal of ovarian remnants in dogs was feasible and effective. The ability to change the position of the patient during the procedure, tilting sideways and in steep Trendelenburg position, is a requirement for proper access to both pedicle areas. A VSD facilitated good tissue dissecting and excision ability. The high success rate of this approach makes laparoscopic treatment of ORS a valid and appealing alternative to open laparotomy.

## Additional file

Additional file 1: Short- and long-term follow-up questionnaire forms that were used to collect follow-up data during telephone contact moments with patient owners. (PDF 104 kb)

## Abbreviations

E_2_: Estrogen
IQR: Inter quartile range
ORS: Ovarian remnant syndrome
OVE: Ovariectomy
OVH: Ovariohysterectomy
P_4_: Progesterone
Sd: Standard deviation
VSD: Vessel sealing device
