# Supplementary material for: A laparoscopic approach for removal of ovarian remnant tissue in 32 dogs
Source: BMC Vet Res. 2018 Nov 7;14:333. doi: 10.1186/s12917-018-1658-y (PMC6223097; doi:10.1186/s12917-018-1658-y)
Supplement: Supplementary file 1 — Short- and long-term follow-up questionnaire forms that were used to collect follow-up data during telephone contact moments with patient owners. (PDF 104 kb) [file 12917_2018_1658_MOESM1_ESM.pdf]

# A laparoscopic approach for removal of ovarian remnant tissue in 32 dogs

## - Additional file: follow-up questionnaire form -

Sebastiaan A van Nimwegen[1], DVM, PhD, DECVS, Bart Van Goethem[2], DVM, PhD, DECVS, Jeffrey de Gier[1], DVM, PhD, DECAR, Jolle Kirpensteijn[1,3], DVM, prof, PhD, DECVS&DACVS

[1]Department of Clinical Sciences of Companion Animals, Faculty of Veterinary Medicine, Utrecht University, Yalelaan 108, 3584CM, Utrecht, the Netherlands

E-mail: S.A.vanNimwegen@uu.nl; J.deGier@uu.nl; Jollenl@gmail.com

[2]Department of Small Animal Medicine, Faculty of Veterinary Medicine, Ghent University, Salisburylaan 133, 9820, Merelbeke, Belgium

E-mail: Bart.VanGoethem@ugent.be

[3]Hill's Pet Nutrition, 400 SW 8th Ave, Topeka, KS, 66603, USA

E-mail: jollenl@gmail.com

Corresponding author: S.A. van Nimwegen

**Follow-up questionnaire form after surgery for ORS** (modified from digital version in hospital patient data system)

Patient details: Patient number, owner name and contact data

Date of surgery: ……………………..

## Short-term follow-up

Date of follow-up: ……………………..

Appetite: □ decreased □ normal □ increased

Exercise level: □ decreased □ normal □ increased

Surgical wounds

| | | | | |
|---|---|---|---|---|
| Wound 1 | □ normal | □ irritated | □ swollen | □ open/discharge |
| Wound 2 | □ normal | □ irritated | □ swollen | □ open/discharge |
| Wound 3 | □ normal | □ irritated | □ swollen | □ open/discharge |

Recovery [days] □ 0,5 □ 1 □ 2 □ 3 □ 4 □ 5 □ 6 □ 7

pain medication [days] □ 1 □ 2 □ 3 □ other: ……………………………………………

Signs of pseudopregnancy: (behavior/swollen nipples, vulva/other)

……………………………………………………………………………………………..

……………………………………………………………………………………………..

Other clinical signs or remarks: ………………………………………………………………………………………………

………………………………………………………………………………………………

………………………………………………………………………………………………

Owner satisfaction: □ not satisfied □ satisfied □ very satisfied

**Intermediate/long-term follow-up**

Date of follow-up: ……………………. (Form can be repeated with new date)

General health status: ……………………………………………………………………………………………….

……………………………………………………………………………………………….

Appetite: □ decreased □ normal □ increased

Exercise level: □ decreased □ normal □ increased

Signs of ORS □ attractiveness to other dogs: ……………………………………………..

□ vulva discharge, aspect: ………………………………………………

□ swollen vulva and/or nipples: ………………………………………………

□ other signs or behavior: ………………………………………………

……………………………………………………………………………………………….

………………………………………………………………………………………………

Other clinical signs or remarks: ………………………………………………………………………………………

………………………………………………………………………………………

………………………………………………………………………………………
